# Extraction and characterization of a newly developed cellulose enriched sustainable natural fiber from the epidermis of *Mikania micrantha*

**DOI:** 10.1016/j.heliyon.2023.e19360

**Published:** 2023-08-22

**Authors:** Fahmida-E- Karim, Md. Redwanul Islam, Rizbi Ahmed, Abu Bakr Siddique, Hosne Ara Begum

**Affiliations:** a Department of Textile Engineering, BGMEA University of Fashion and Technology (BUFT), Dhaka, Bangladesh; b Department of Textile Engineering, Ahsanullah University of Science and Technology (AUST), Dhaka, Bangladesh; c Department of Yarn Engineering, Bangladesh University of Textiles (BUTEX), Dhaka, Bangladesh

**Keywords:** Sustainable natural fiber, Cellulose, *Mikania micrantha*, Retting

## Abstract

Riding on the journey of a sustainable world it is very crucial to extend the usage of natural cellulosic fiber from renewable sources. Due to their numerous applications and eco-friendly behavior, natural cellulosic fibers are in greater demand every day. In this article a new natural fiber extracted from the creepers of *Mikania micrantha* with the help of 5% NaOH retting process. Previously no research work have been done with this fiber. The fiber was characterized by following ASTM D1909, ASTM D 2654, ASTM D1445, TAPPI standard for determination of moisture regain and content, bundle fiber strength and chemical composition respectively. XRD, SEM, FTIR and TGA analysis were also done for the identification of crystallinity, fiber morphology, functional group and thermal behavior. The tests results showed that it is a cellulose enriched textile fiber having 56.42% cellulose. The average moisture regain and content % were 9.17% and 8.4% respectively analyzed from the five samples. The average tenacity was determined 38.6 gm/tex with 1.8% elongation and the crystallinity of the tested fiber was 72%. The maximum degradation temperature for this fiber was 477 °C. The application of this noble fiber can be for making fiber reinforced composites, cellulose nanomaterials, biomaterials etc.

## Graphical abstract

**Figure:**
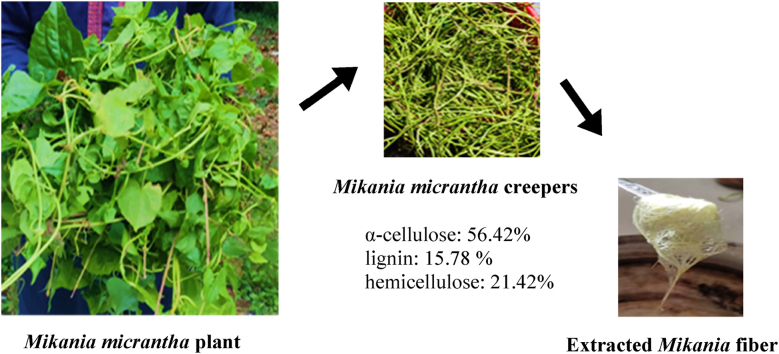

## 1 Introduction

Natural fibers that contribute positively to sustainability are those that are collected and used in a way that minimizes their adverse effects on the environment and society [1]. These fibers are often manufactured using socially responsible methods that guarantee fair labor conditions and social welfare. They also typically have low environmental impacts in terms of resource use, energy use, and pollution [2]. Because they are biodegradable, have a reduced carbon impact, and have the potential to be used in circular economy processes, sustainable natural fibers are frequently chosen over synthetic fibers [2]. Natural cellulosic fiber has seen a steady increase in popularity in recent years, and researchers in both academia and business are now actively involved in investigating novel natural fibers and their potential applications [3,4]. The primary cause of this interest is related to their unique features that are appropriate for a variety of complex fibrous applications, including reinforcement in composite, textile, cellulose nanomaterials, activated or conductive carbon, biomaterials, etc. [1]. Natural fibers have special qualities including low density, low price, availability, recyclability, non-toxicity, significant strength, high thermal stability, biodegradability, etc. [5]. The name of natural fibers in traditional and recent are flax, hemp, jute, kapok, coir, ramie, pineapple, cotton, bagasse etc. [6].

Sustainable natural fibers are used to create apparel, textiles, and home furnishings, including organic cotton, hemp, bamboo, and linen. In comparison to traditional fibers, these fibers are more ecologically friendly since they are cultivated without the use of dangerous chemicals, genetically modified organisms (GMOs), or excessive water usage [3,4,7]. Additionally, they are biodegradable, which lessens the environmental impact of their life cycle. These natural fibers can be used in place of materials based on plastic in packaging [8]. In order to create biodegradable bags, sacks, and packaging materials, for instance, jute, a natural fiber generated from the jute plant, can be utilized [1]. These fibers also can be used for construction purposes. For instance, flooring, wall panels, and furniture may all be made from bamboo, a quickly growing and reusable natural fiber [8]. Paper and cardboard may be made from sustainable natural fibers like hemp, kenaf, and flax. It is hard to list all the usage of sustainable natural fibers. As a result of their high demand in fibrous applications, it is crucial to investigate novel natural sources of cellulosic fiber [9].

A fast-growing vine that is indigenous to Central and South America is *Mikania micrantha*. It was purposefully introduced into a number of nations; since then, it has established itself as a significant weed throughout Southeast Asia and the Pacific, and it is continuously expanding [10]. A natural remedy for treating fever, rheumatism, colds, respiratory illnesses, and snakebites is Mikania micrantha [10], also called “Libujilota/Germany lota” in Bangladesh [11]. The use of it as a folk remedy to treat ulcers and enhance wound healing is also quite common in Africa. Deoxy Mikanolide, scandenolide, dihydroscandenolide, mikanolide, dihydromikanolide, and *m*-methoxybenzoic acid are the components of M. micrantha that give it its antibacterial characteristics [12]. Due to its extensive range of bioactivities, which include anticancer, antifungal, cytotoxic, analgesic, antimicrobial, antioxidant, and antiviral, it is utilized widely outside of its native Central and South America and Southeast Asian regions [10,12]. Compared to this fiber, some fast growing vines that are planted in Central and South America are Bougainvillea, Carolina Jessamine, Clematis, Climbing Hydrangea, Creeping Fig, Honeysuckle, Morning Glory etc. [13]. The aim of this paper is to collect the fibers from the bark of *Mikania micrantha* and characterize it and analyze its property to introduce it as a sustainable natural fiber. A simple research work had been done on this fiber where the chemical and natural extraction was done for finding the properties like slenderness ratio, flexibility ratio and Runkel ratio of *Mikania micrantha* [14]. Apart from this no investigation was found that related to this work which indicated the novelty of this research.

## 2 Experimental

### 2.1 Fiber extraction process

**Fig. 1:**
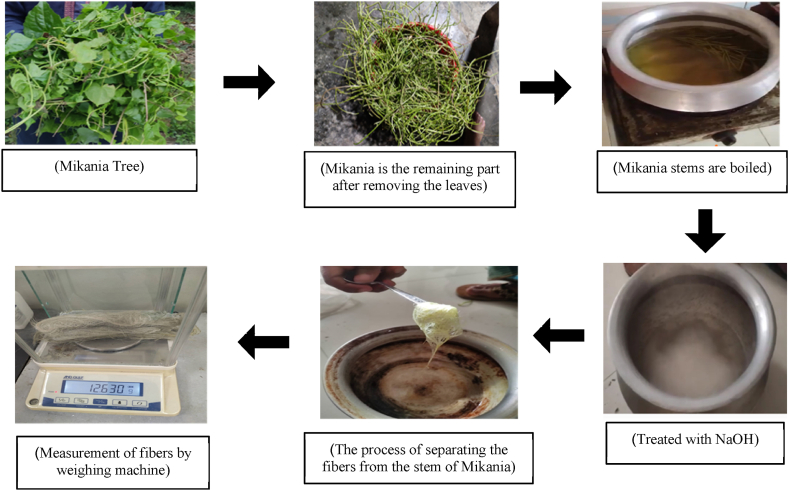
*Mikania* fiber extraction flow chart.

At first Creepers were collected from the roadside of the village of Narail, Bangladesh. Then leaves were cut from the creeper. After that all the creepers were cut into pieces. Sodium hydroxide (NaOH) was used as a retting solution to remove fibers from the stems of *Mikania micrantha*. *Mikania micrantha* stems were immersed in a 5% NaOH solution for 15–20 min in a 90–100 °C water bath. Strong alkali conditions, along with high temperatures, caused soft tissue degeneration and fiber separation. To eliminate any leftover tissues, the separated fibers were rinsed under running water and neutralized with acetic acid. By repeating the trials, suitable temperature conditions were established. Following completion of this operation, the final fiber is collected in the needed quantity. The process was given in the below Fig. 1.

### 2.2 Fiber characterization methods

#### 2.2.1 FTIR analysis

A Fourier Transform Infrared (FTIR) Spectrophotometer (FT-IR 8400 S, Shimadzu Corporation, Japan) was used to determine the functional compound of the fiber. The fibers were crushed and blended with potassium bromide (KBr) to measure the infrared of the fibers because KBr is transparent. In absorbance mode, the scan rate of the FTIR spectrometer was 32 with a resolution of 2 per cm in the wave number region of 400–4000 cm^−1^ at a room temperature of 30 °C and RH of 65%.

#### 2.2.2 Moisture regain and content analysis

The ASTM D1909 [15], ASTM D 2654 [15] technique was used to determine the moisture regain and content percentage respectively. A 5-g sample was obtained under conventional fiber testing conditions of 20 °C and 65% relative humidity. The weighted fiber samples were put in an air oven at 105 °C for a consistent time. Using the equation below, the moisture content percentage of fiber was computed.

$$\frac{W}{D}\times 100\%=MR\%$$

$$\frac{W}{W+D}\times 100\%=MC\%$$

Here,W= Weight of water D = Oven dry weight MR= Moisture regain MC= Moisture content.

#### 2.2.3 Bundle fiber strength analysis

Bundle fiber strength test was done by using Stelometer. The test method is ASTM D1445 [16]. The well combed fiber samples from the eight different types of fibers were placed on the pair of small clamp where the gauge length was zero. After inserting the fiber clamp at the top of the pendulum, fibers were broken by giving tension. From the process, breaking strength and elongations of the fibers were determined.

#### 2.2.4 XRD analysis

X-ray diffractometer (Smart Lab SE, Regaku, Japan) with X-ray generator settings tings of 50 mA current and 40 kV voltages was used to assess the fiber's crystallinity. The X-ray diffraction investigation was performed on the fiber pellet sample. CuKα (1.54 Å) radiation's diffraction intensity was measured between 10 ͦ and 40 ͦ (2β angle range) using a scanning speed of 15 ͦ/min and a step width of 0.01 ͦ. The accompanying Segal empirical equation [17] was used to construct the peak height approach for calculating the crystallinity index (CI) in the cellulosic materials.

$$CI=\frac{I_{200}-I_{am}}{I_{200}}$$

#### 2.2.5 TGA and DSC analysis

DSC examination was carried out on fibers using a thermal analyzer. (TA Instruments Trios V5.1.1.46572, New Castle, DE, USA). Between 10 °C and 450 °C, at a rate of 10 °C/min, fiber samples (weighing 10 mg) were heated. The experiment was carried out in a nitrogen atmosphere that was being purged at 100 ml min^−1^. Using an aluminum pan and a thermogravimetric analyzer (TA Instruments Trios V5.1.1.46572, New Castle, DE, USA), the thermal stability of the fiber was tested at a heating rate of 10 ͦ C min^−1^ and a flow rate of 100 mL min^−1^. Dry material weighing 10.39 mg was utilized in this experiment. The thermal analyzer's test chamber temperature was raised progressively from 32.5 C to 477.5 ͦ C at a rate of 10 ͦ C per minute.

#### 2.2.6 SEM analysis

To investigate the morphological characteristics of fiber interfaces The SEM image was acquired at the following settings: 10 KV accelerating voltage; secondary electron image mode; 20 mm working distance; and 500X magnification. Using the same microscope, the surface morphologies of fiber samples before and after chemical treatments were examined. The model number of the machine was HITACHI SU1510.

#### 2.2.7 Chemical composition analysis

According to TAPPI standard test techniques, the chemical composition [18] of the fiber extracted from the bark of *Mikania micrantha* creepers, including its cellulose, hemicellulose, lignin, extractive, and ash contents, were measured. By employing the Soxhlet extraction method and a solvent solution of ethanol and toluene, the extractive percentage was calculated. (TAPPI T204 om-88). Utilizing a NaClO_2_ solution, extractive free fibers were utilized to make holo-cellulose, which was then treated with a NaOH solution to determine its -cellulose content (TAPPI T203 om-93). The Klason approach was used to calculate the proportion of lignin applied (TAPPI T211 om-83). According to the TAPPI (T211 os-76) test technique, the ash content was assessed [19]. There were two replications. During each compositional analysis, the average is taken into consideration.

## 3 Results and discussion

### 3.1 FTIR analysis

**Fig. 2:**
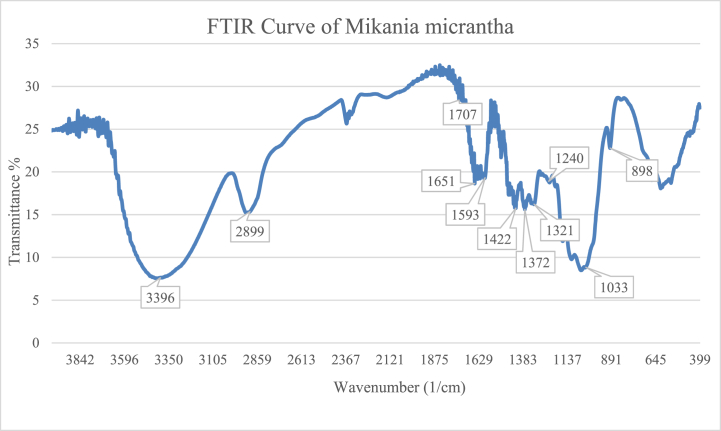
FTIR graph for *Mikania micrantha* fiber.

Mikania micrantha fiber's FTIR spectrum is shown in Fig. 2. Peak locations for the fiber were recorded along with the matching chemical functional group designations represented in Table 1.

**Table 1:** Chemical stretching distributions and peak locations in FTIR for *Mikania micrantha* fiber.

Peak Locations wavenumber (cm^−1^)	Allocations	Reference
3396	Broad peak for H bonded stretching vibration of –OH bond	[20]
2899	C–H stretching vibration for cellulose and hemicellulose	[21]
1707	C]O (carbonyl groups) of lignin and hemicellulose	[3,21]
1651	Strong peak for the water absorption of natural cellulose	[[22], [23], [24]]
1593	Low intensity peak for C]C groups in lignin	[25]
1422	Bending vibration of cellulosic CH_2_ groups	[24,25]
1372	C–H vibration	[26]
1321	Due to O–H bending	[20,27,28]
1240	Acetyl group vibration in lignin with C–O stretching	[29]
1033	C–OH vibration	[20,26]
898	Β-glycoside linkage	[27]

There were distinct peaks in the spectra at 3396, 2899, 1707, 1651, 1593, 1422, 1372, 1321, 1240, 1033 and 898. From the functional group region the stretching vibration of the hydrogen-bonded hydroxyl group had a large peak at 3396 cm^−1^, which corresponded –OH to it. The peak for C–H stretching vibration of cellulose and hemicellulose is at 2899 cm^−1^ [21]. According to several sources [3,21] the carbonyl groups (C]O) of lignin and hemicellulose were represented by the peaks at 1707 cm^−1^. Natural cellulose fiber had a significant peak in water absorption at around 1651 cm^−1^ [[22], [23], [24]]. The C]C groups of lignin were shown by a peak of extremely low intensity at 1593 cm^−1^. The C–H bending vibration was linked to the occurrence of the peak at 1372 cm^−1^. The O–H bending causes the peak at 1321 cm^−1^ [26]. In lignin, the acetyl group had a C–O stretching vibration at 1240 cm^−1^. Peaks in cellulose and hemicellulose concentration at the fingerprint region from 1100 to 1200 cm^−1^ were ascribed to C–O–C vibration. The peak at 1033 cm^−1^ was thought to be caused by C–OH vibration, while 896 cm^−1^ was related to -glycoside linkage [20,26,27]. The FTIR peak patterns confirmed this fiber's high cellulose content [21]. In lignin, the acetyl group had a C–O stretching vibration at 1240 cm^−1^. Peaks for cellulose and hemicellulose at around 1100–1200 cm^−1^are due to C–O–C vibration.

### 3.2 Moisture regain and content analysis

The quantity of water a fiber contains had a significant impact on its composition. The degree of comfort a textile may provide depends heavily on its capacity to retain body heat in a variety of climatic conditions. In this aspect, moisture management is a crucial performance component. Changes in moisture content have an impact on the tensile strength, elasticity, fiber diameter, and friction of textiles. A reduction in a textile's equilibrium relative humidity might make it more fragile, brittle, and weak. By maintaining air humidity during processing the fibers, this moisture loss to the atmosphere is reduced to a minimum [23]. Table 2 depicted the chart of moisture regain and moisture content percentage of the samples.

**Table 2:** Calculating moisture regain and content percentage for *Mikania* fiber.

Sample No.	Sample Weight	Oven dry Weight of Sample(g)	Weight of Water(g)	Moisture Regain (%)	Average Moisture Regain (%)	Moisture Content (%)	Average Moisture Regain (%)
Sample 1	5.0	4.59	0.41	8.9	9.17	8.2	8.4
Sample 2	5.0	4.57	0.43	9.4		8.6	
Sample 3	5.0	4.58	0.42	9.17		8.4	
Sample 4	5.0	4.60	0.40	8.69		8.0	
Sample 5	5.0	4.56	0.44	9.64		8.8	
Standard Deviation				0.33		0.28	
Co-efficient of variation				3.7%		3.36%	

**Fig. 3:**
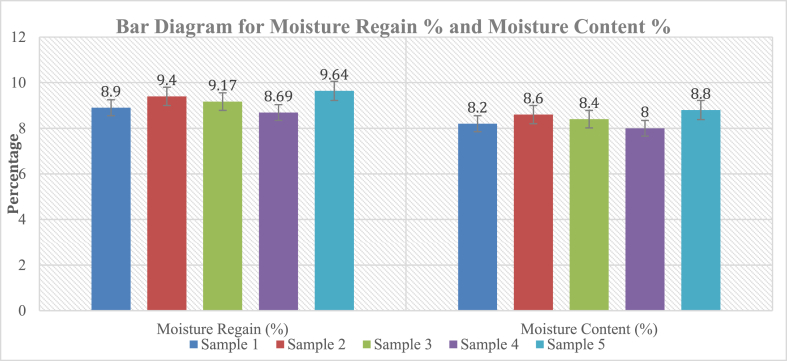
Bar diagram for moisture regain and content of *Mikania micrantha* fiber.

From the investigation, it was found that the average moisture regain and content is 9.17% and 8.4%. The result was collected from five specific samples 5 gm each from the extracted fibers. The standard deviation of this two results 0.33 and 0.28 respectively showed that the fiber samples had nearly close values for moisture regain and moisture content. The bar diagram in Fig. 3 depicted the error values moisture regain % on the left side and moisture content% on the right side for the five tested samples.

### 3.3 Bundle fiber strength analysis

Strength is one of a textile fiber's key characteristics. Strength, a metric for a material's limited load bearing capability, is determined by the load that it can support before breaking. Weak fibers cannot be spun into a strong yarn. The fiber strength must be taken into account before being used as a reinforcing element. The stelometer test showed the bundle fiber strength. The data found by testing *Mikania micarntha* is enlisted Table 3.

**Table 3:** Calculating breaking force, tenacity and elongation for *Mikania micarntha* fiber.

Properties	Breaking force (kg)	Tenacity (gm/tex)	Elongation %
Sample 1	4.1	41	1.8
Sample 2	3.9	36	1.8
Sample 3	4.15	37	1.8
Sample 4	4.2	38	1.8
Sample 5	4.1	41	1.8
Average value	4.09	38.6	1.8
Standard Deviation	0.09	1.88	0.00
Co-efficient of variation	2.28	4.87	0.00

**Fig. 4:**
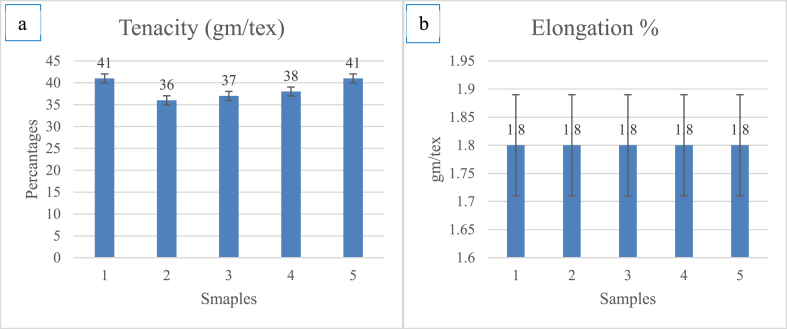
Bar diagram for a. Tenacity and b. Elongation % of *Mikania micarntha* fiber.

From the data Tables 3 and it was found that the average tenacity of *Mikania micarntha* fiber was 38.6 gm/tex with 1.8% elongation. As it is a natural fiber the strength can vary from sample to sample. The coefficient of variation for its tenacity was investigated 4.87% from the selected five samples. The bar diagram showed the error values of the tested samples from the stelometer results. Here Fig. 4(a) showed the bar diagram and error values for fiber tenacity in gm/tex and Fig. 4(b) indicated the bar diagram and error values for fiber elongation% respectively.

### 3.4 XRD analysis

The main construction of cellulose are observed from nature, and is constituted with 2 types of cellulose that is I_α_ and I_β_. In plant fibers I_β_ is more available than the other one for example cotton, jute, flax and hemp etc. [30]. Beta cellulose forms by removing water and making bridges of C-1 and C-4 oxygen. In this case a van der Waals contact stabilizes the stacking of paralleled hydrogen-bonded sheets [31].

**Fig. 5:**
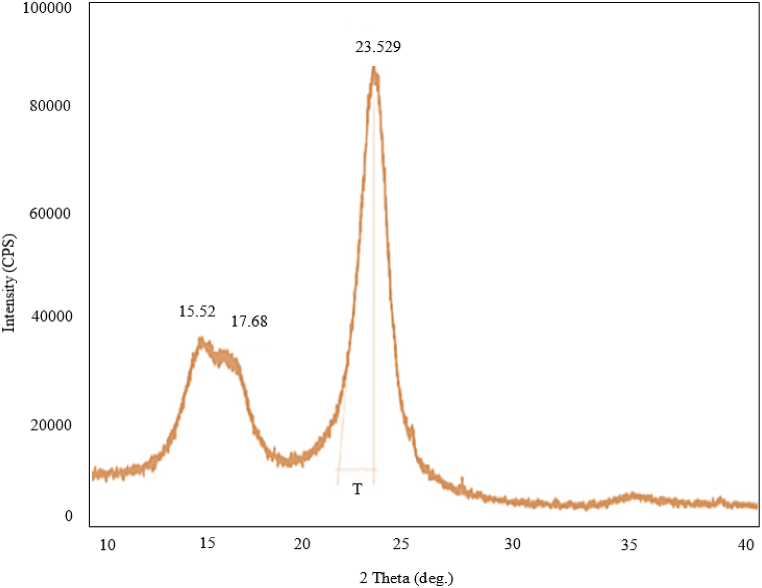
XRD diagram for *Mikania micarntha* fiber.

Fig. 5 displays X-ray diffractograms of *Mikania micarntha*. The main crystalline peak of *Mikania micarntha* was seen at 2θ = 23.529, and well-defined peaks are evident at 2θ = 15.52 linked to the usual diffraction of cellulose-I. However, the fiber has a greater proportion of amorphous components (lignin, hemicellulose, and amorphous cellulose), which is why the least intensity peaks were detected at 2θ = 17.68. The peak was more noticeable and the fiber was encased in a substantial amount of amorphous material, such as lignin, hemicelluloses, and amorphous cellulose as the crystalline cellulose percentage (CI = 72.01%) is high in the fibers [32]. Table 4 showed the comparison of the well-established natural fibers CI with the tested one.

**Table 4:** Comparative study for different natural fibers chemical compositions with *Mikania micarntha*.

Name of the fibers	Cellulose %	Hemi-cellulose %	Lignin %	Crystallinity index	Extraction process	references
*Mikania micrantha*	56.42	21.42	15.78	72	5% NaOH, 90–100 ͦ C	Current work
Jack tree fiber	79.32	8.01	6.77	86	Water retting	[20]
Jute	64.4	12	11.8	71	Water retting	[26]
Hemp	67–75	16–18	3–5	88	–	[33]
Flax	62–71	16–18	3–4.5	80	–	[33]
Cornhusks	80–87	–	–	48–50	0.5 N NaOH, 95ͦ C	[34]
Napier grass	45.66	33.67	20.60	–	chemical retting	[22]
sugar cane straw	33.5	–	25.8	–	–	[29]
Typha australis	35.83	21.42	17.60	–	chemical retting	[35]
Star jasmine	62.7	14.5	7.6	87.68	Water retting and then Enzyme retting	[33]
Cotton stalk	79	[	13.7	47	2 N NaOH, boiling temperature	[36]
Sorghum stems	65	–	6.5	2% NaOH, 95 °C	–	[37]
Albizia amara	64.54	14.32	15.61	63.78	Water retting	[24]
Acacia Planifrons	73.1	9.41	12.04	65.38	Water retting	[24]
Furcraea foetida	68.35	11.46	12.32	52.56	Water retting	[28]

### 3.5 TGA and DSC analysis

**Fig. 6:**
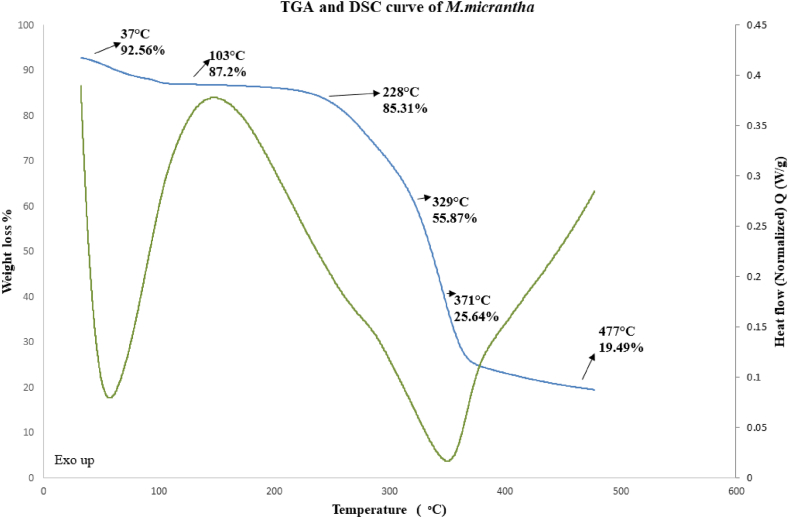
TGA & DSC analysis diagram for *Mikania micarntha* fiber.

The below Fig. 6 depicted the thermogravimetric behavior of the fiber sample. Due to the evaporation of water from the surface at around 100 ͦ C the change of mass was found 13%. Then the fiber remained thermally stabled with 1.8% mass loss only at 100 ͦ C to 228 ͦ C. Basic three elements that controls the thermal stability are lignin, cellulose and hemicellulose. Among these three, lignin is more stable than hemicellulose and cellulose. The maximum degradation was happened after 228 ͦ C where hemicellulose components were responsible it. At 371 ͦ C about 75% fiber weight was degraded as a result of cellulose degradation. Rest of the degradation was occurred by the involvement of lignin and the measurement was done up to 477 ͦ C where around 90% degradation took place. Hemicellulose has a significant impact on a fiber's moisture content and first thermal breakdown behavior [38]. The hemicellulose-rich natural fibers are more able to absorb moisture and deteriorate at a lower temperature. In a similar manner, large amounts of extractives can speed up fiber breakdown at lower temperatures. As a result, based on their chemical makeup, natural fibers' deterioration trend may be estimated. The examined fiber had higher hemicellulose than the other fibers so it sustained up to 477 ͦ C which was comparatively lower than the jackfruit fiber [20].

As the fibers heat up, differentiating scanning calorimetry analysis is able to show the changes that take place in them and detect their phase transformation. The molecules' volatilization (to gas) caused the endothermic reaction to occur, whereas the creation of char (to solid residue) caused the exothermic reaction [39]. Due to the evaporation of moisture, an endothermic peak under 100 ͦ C was observed. The maximum stable state of the fibers is represented by the peaks in the temperature range between 150 and 280 °C, which do not exhibit any endothermic or exothermic processes.

A comparison Table 5 for the thermal behavior of different fibers enlisted below that visualized the compatibility of *Mikania micarntha* with the other fibers.

**Table 5:** Comparison list for the thermal behavior of different natural fibers.

Fiber Name	Elementary decomposition temperature (°C)	Eventual decomposition temperature (°C)	Reference
*Mikania micarntha* fiber	103 °C	477 °C	This work
*Phaseolus vulgaris* fiber	91.2 °C	328.5 °C	[40]
*Prosopis juliflora* fiber	66.2 °C	351.3 °C	[41]
*Azadirachta indica*	97 °C	321.2 °C	[42]
Jack tree fiber	104.45 °C	565 °C	[20]
*Heteropogon Contortus*	90.1 °C	337.7 °C	[43]
*Saccharum Bengalense* Grass	100 °C	600 °C	[44]

### 3.6 SEM analysis

**Fig. 7:**
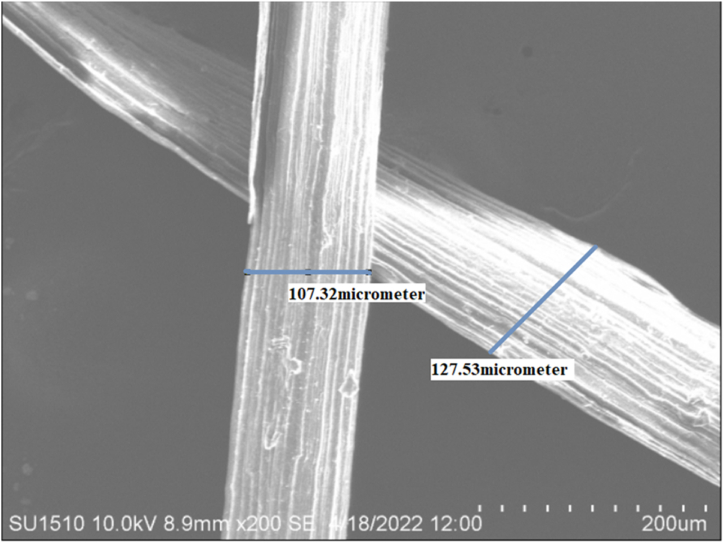
SEM image of *Mikania micarntha* fiber.

**Fig. 8:**
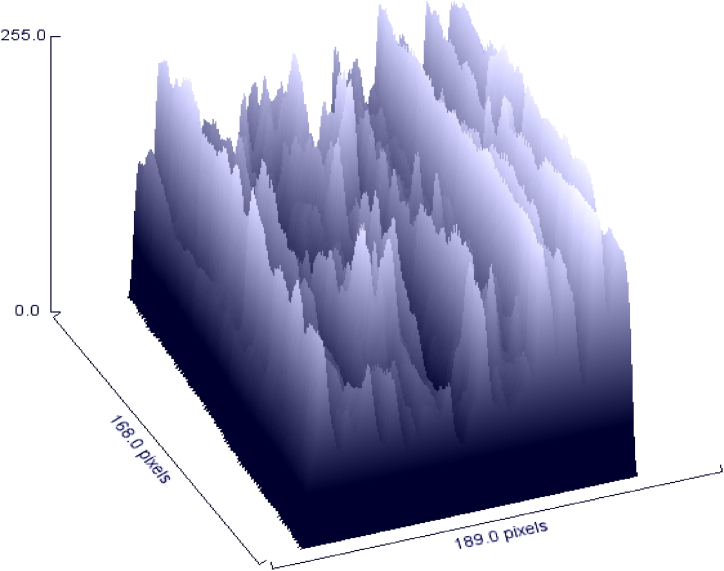
Surface roughness of *Mikania micarntha* fiber.

The quality of the fibers was assessed using scanning electron microscopy, which enables determining whether scale, thickness, or irregularities are present along the fibers. In the medical industry, SEM may be used to assess the presence of microorganisms such as fungus, bacteria, and cell adhesion to the material surface [29]. The morphological view appearing in Fig. 7 was denoting the uneven and rough surface of *Mikania micarntha*. The fiber diameter varied from one to another. The fiber diameter was found to be 110 μm on average. Lignin and hemicellulose, which are known as the fiber's surface binding substances, make up the outer layer of the fiber. In order to provide greater adhesion of the fiber with the matrix in composite applications, the surface roughness leads to increased contact surface area [44]. The creepers were cut into small pieces that made the fiber length small. Fig. 8 denoted the fluctuations of the fiber surface that represented the harsh surface of this fiber. For using this in any composite making purpose the length can be adjusted by cutting it from the plant. Fig. 8 established the high amount of surface roughness of this tested fiber which made it more compatible for adhesion.

### 3.7 Chemical composition analysis

Chemical composition identification is essential for any kind of material to decide its chemical behavior. The characteristics, structure, qualities, and process ability of fiber are influenced by its content. The *Mikania micrantha* fiber was found to be enriched with α-cellulose, lignin and hemicellulose. The percentage of α-cellulose was 56.42, lignin percentage was 15.78 and hemicellulose percentage was 21.42. High cellulose content promotes high value applications and makes it reasonably simple to process them for a variety of uses. It can improve a material's crystalline characteristics, hydrolysis resistance, thermal stability, biodegradability, mechanical properties, and other qualities. Hemicellulose and lignin concentration in moderate amounts present any fiber can help to manage the fiber bundle and stiffness, making them appropriate for high-value composite applications. The moisture content was found 8.4%, ash and extractive percentage were found 1.9% and 1.1% respectively.

## 4 Conclusion

Cellulose is the most abundant textile raw material which is used widely in different purposes like clothing, industry applications, medical textile, fiber reinforced composite materials and so on. Researchers have always stayed focused on finding the new sources of cellulose. The article also introduced and characterized a new cellulosic fiber that is *Mikania micrantha*. 5% NaOH used for the retting process and then the sample was characterized. Its chemical composition reveals its enriched cellulose (56.42%). The fiber and cotton are comparable, according to XRD and FTIR measurements. By using SEM examination, good morphology was found. The fiber is thermally stable up to 228 °C, according to thermogravimetric analysis and derivative thermogravimetric (DTG), and the main degrading temperature range is between 228 °C and 329 °C. The cellulose content and crystalline structure of the fiber are indicated by this breakdown pattern. This fiber has a fair degree of elongation and a moderate amount of strength. The suitability of using this fiber as reinforcement for bio-composite materials, cellulose nanomaterials, and biomaterials is currently the subject of additional investigation.

## Funding statement

This research did not receive any specific grant from funding agencies in the public, commercial, or not-for-profit sectors.

## Author contribution statement

Fahmida-E− Karim: Conceived and designed the experiments; Wrote the paper. Md. Redwanul Islam: Hosne Ara Begum: Analyzed and interpreted the data. Rizbi Ahmed: Performed the experiments. Abu Bakr Siddique: Contributed reagents, materials, analysis tools or data.

## Data availability statement

Data will be made available on request.

## Additional information

No additional information is available for this paper.

## Declaration of competing interest

The authors declare that they have no known competing financial interests or personal relationships that could have appeared to influence the work reported in this paper.
